# The number of polyploid giant cancer cells and epithelial-mesenchymal transition-related proteins are associated with invasion and metastasis in human breast cancer

**DOI:** 10.1186/s13046-015-0277-8

**Published:** 2015-12-24

**Authors:** Fei Fei, Dan Zhang, Zhengduo Yang, Shujing Wang, Xian Wang, Zhengsheng Wu, Qiang Wu, Shiwu Zhang

**Affiliations:** Department of Pathology, Anhui Medical University, Hefei, Anhui 230032 People’s Republic of China; Department of Pathology, Tianjin Union Medicine Center, Tianjin, 300121 P.R China

**Keywords:** Polyploid giant cancer cells, Breast cancer, Epithelial-mesenchymal transition, Metastasis

## Abstract

**Background:**

Previously, we reported that polyploid giant cancer cells (PGCCs) induced by cobalt chloride (CoCl_2_) could have generated daughter cells with strong invasiveness and migration capabilities via asymmetric divisions. This study compared the expression of epithelial-mesenchymal transition (EMT)-related proteins, including E-cadherin, N-cadherin, and vimentin, between PGCCs and their daughter cells, and control breast cancer cell lines MCF-7 and MDA-MB-231. The clinicopathological significance of EMT-related protein expression in human breast cancer was analyzed.

**Methods:**

Western blot was used to compare the expression levels of E-cadherin, N-cadherin, and vimentin in breast cancer lines MCF-7 and MDA-MB-231, between PGCCs with budding daughter cells and control breast cancer cells. Furthermore, 167 paraffin-embedded breast tumor tissue samples were analyzed, including samples obtained from 52 patients with primary breast cancer with lymph node metastasis (group I) and their corresponding lymph node metastatic tumors (group II), 52 patients with primary breast cancer without metastasis (group III), and 11 patients with benign breast lesions (group IV). The number of PGCCs was compared among these four groups.

**Results:**

The number of PGCCs increased with the malignant grade of breast tumor. Group IIhad the highest number of PGCCs and the differences among group I, II, III and IV had statistically significance (*P* =0.000). In addition, the expression of E-cadherin (*P* = 0.000), N-cadherin (*P* = 0.000), and vimentin (*P* = 0.000) was significantly different among the four groups. Group II exhibited the highest expression levels of N-cadherin and vimentin and the lowest expression levels of E-cadherin.

**Conclusions:**

These data suggest that the number of PGCCs and the EMT-related proteins E-cadherin, N-cadherin, and vimentin may be valuable biomarkers to assess metastasis in patients with breast cancer.

**Electronic supplementary material:**

The online version of this article (doi:10.1186/s13046-015-0277-8) contains supplementary material, which is available to authorized users.

## Background

Breast carcinoma is the most common cancer and the leading cause of cancer death in women around the world, accounting for 29 % of total new cancer cases in women and the most common causes of cancer death are cancers of the lung and bronchus, breast, and colorectum in women in 2015 [1]. Breast cancer is the leading cause of cancer death in women aged 20 to 59 years [1]. Recurrence and metastasis of breast cancer after surgical removal of the primary tumor are the leading causes of death in breast cancer patients, while present anti-tumor treatment has improved the 5-year survival rate of patients with breast cancer [2]. Currently, the detailed mechanisms of metastasis in breast cancer are complicated, and many proteins and signaling pathways are involved in the process of cancer metastasis.

Recently, we have reported that polyploid giant cancer cells (PGCCs) induced by cobalt chloride (CoCl_2_) are a key contributor to cancer occurrence, development, invasion, metastasis, and chemoresistance [3]. PGCCs are a special sub-population of cancer cells, and the nuclei of PGCCs are usually irregular. PGCC nuclei are at least three times greater in size than regular-sized diploid tumor cell nuclei [4]. They promote the heterogeneity of solid tumors and have the properties of cancer stem cells [5, 6]. However, the detailed mechanisms of PGCC formation and their relationships with tumor initiation and metastasis are not completely defined. CoCl_2_ is a hypoxic mimic and can induce the formation of PGCCs by selectively killing regular diploid cells; flow cytometry and fluorescence in situ hybridization (FISH) reveal the presence of multiple copies of DNA in single PGCC [7]. We have successfully isolated, purified, and cultured PGCCs from 22 cancer cell lines, including HEY, SKOV3, and MDA-MB-231 [8]. PGCCs express normal and cancer stem cell markers, and can be induced to differentiate into other tissues, such as adipose, cartilage, erythrocytes, fibroblasts, and bone [3, 4, 9, 10]. In addition, they generate daughter cells (regular-sized diploid cancer cells) via asymmetric cell divisions, a process of reductive division known as depolyploidization [11, 12]. Asymmetric cell division, including splitting, budding, and burst-like, usually occurs in the division of low-level eukaryotes, plants, and viruses [3]. Compared to diploid cancer cells, PGCCs with budding daughter cells and PGCCs alone express lower levels of cytokeratin and higher levels of vimentin, indicating that PGCCs and their budding daughter cells have undergone epithelial-mesenchymal transition (EMT) [10].

EMT has been found to play an important role in cancer development and progression. Cancer initiation and progression are complicated processes that are regulated by a variety of cellular and signaling proteins, and often result from malignant epithelial clones that expand as a result of activating mutations of oncogenes or inactivating mutations of tumor suppressor genes. EMT facilitates the metastasis of several types of human cancer, such as bladder cancer, primary liver cancer, and malignant melanoma [13]. The concept of EMT was first put forward by Greenberg et al. [14]. Now, more and more evidence confirms that EMT plays an important role in the process of cancer metastasis [15]. After epithelial cancer cells undergo EMT, they lose epithelial characteristics and cell polarity instead of gaining mesenchymal characteristics, and highly express EMT markers, including snail, slug, and Twist [16]. In addition, the epithelial marker, E-cadherin, and the mesenchymal markers, N-cadherin and vimentin, are regarded as important markers of EMT [17–19], and are widely used in invasion and metastasis cancer research. It is reported that many cancer cells exhibit decreased expression of E-cadherin and increased expression of N-cadherin and vimentin, which are significantly correlated with the malignant degree of cancers and the metastasis of cancers to the lymph nodes. These alterations are in agreement with the EMT phenotype reported in breast cancer [19, 20] and esophageal squamous cell carcinoma [21].

PGCCs and their budding daughter cells gain strong invasiveness and migration ability after they undergo EMT. This study compared the expression of EMT-related proteins between PGCCs with budding daughter cells and control cells without CoCl_2_ treatment. We also determined the clinicopathological significance of EMT-related protein expression and the number of PGCCs in breast cancer.

## Methods

### Cancer cell lines and culture

The human breast cancer cell lines MCF-7 and MDA-MB-231 were obtained from the American Type Culture Collection (ATCC; Manassas, VA, USA). MCF-7 cells were maintained in complete ATCC-formulated RPMI-1640 medium (HyClone, Logan, UT, USA), and MDA-MB-231 cells were grown in ATCC-formulated Leibovitz's L-15 medium (Gibco, Norwalk, CT, USA), supplemented with 10 % fetal bovine serum (FBS; ExCell Bio, Shanghai, China), 100 U/mL penicillin, and 100 μg/mL streptomycin. Cells were routinely incubated at 37 °C and 5 % CO_2_ under a humidified atmosphere.

### Formation of PGCCs

MCF-7 and MDA-MB-231 cells were cultured in complete medium in T25 flasks until they reached 80–90 % confluence. The cells were cultured with different concentrations of CoCl_2_ (Sigma-Aldrich, St. Louis, MO, USA) for different periods based on their hypoxia-resistance ability. MCF-7 cells were treated with 450 μM CoCl_2_ for 72 h, and MDA-MB-231 cells were cultured with 300 μM CoCl_2_ for 72 h. The cells were then cultured in regular complete medium after being rinsed with phosphate-buffered saline (PBS). The majority of regular-sized cells died following treatment with CoCl_2_, and only scattered PGCCs survived CoCl_2_ treatment. Ten to 15 days after removal of CoCl_2_, the surviving PGCCs started to generate daughter cells via budding. After three or four additional CoCl_2_ treatments (to acquire a sufficient number of PGCCs), the PGCCs cultured in complete medium with newly budding daughter cells (approximately 30 % PGCCs with 70 % budding daughter cells) were used for western blots and later analysis.

### Cell cycle analysis

Cells (0.5 × 10^6^-1 × 10^6^) were collected by trypsinization and washing in cold PBS twice. Ice-cold 75 % ethanol was slowly added to cells while vortexing gently, and cells were fixed overnight at 4 °C. Then, the cells were rinsed with cold PBS three times, centrifuged at 1000 rpm/min for 5 min, and resuspended in 200–500 μL cold PBS. Next, the cell suspension was incubated with 20 μL RNase A (BestBio, Shanghai, China) for 30 min at 37 °C, and then with 400 μL propidium iodide (BestBio) for 30 min at 4 °C in a dark room. Analyses and measurements were performed on an FACSVerse (BD Biosciences, Franklin Lakes, NJ, USA) flow cytometer at the excitation wavelength of 488 nm.

### Western blot analysis

PGCCs (30 %) of MCF-7 and MDA-MB-231 after CoCl_2_ treatment with budding daughter cells (70 %), and control cells without CoCl_2_ treatment were lysed for 30 min on ice with 100–200 μL ice-cold radio-immunoprecipitation assay (RIPA) lysis buffer, and centrifuged at 12,000 rpm/min for 30 min at 4 °C. Protein concentration was determined, and protein were separated on a 10 % sodium dodecyl sulfate (SDS) polyacrylamide gel and transferred to a polyvinylidene fluoride (PVDF) membrane (Beyotime, Haimen, China). After blocking with 5 % nonfat milk in 1× Tris-buffered saline with 0.05 % Tween-20 for 3 h at 20–25 °C, the membranes were incubated with rabbit anti-E-cadherin polyclonal (1:300 dilution; BIOSS, Woburn, MA, USA), rabbit anti-N-cadherin polyclonal (1:300 dilution; BIOSS), rabbit anti-vimentin polyclonal (1:300 dilution; BIOSS), and mouse anti-β-actin monoclonal (1:800 dilution; Zhongshan Inc., Beijing, China) antibodies overnight at 4 °C. Membranes were then incubated with the appropriate dilution of secondary antibodies at 20–25 °C for 2 h. SuperSignal West Femto Substrate (ECL detection kit, Thermo Scientific, Norwalk, CT, USA) was used to detect protein expression. Images were captured on a film processor and the optical density of each protein band detected was analyzed with Image-J software. All western blot experiments were repeated multiple times, with β-actin being used as a protein-loading control.

### Cell migration assay

The migration of control and CoCl_2_-treated MCF-7 and MDA-MB-231 PGCCs with their budding daughter cells was detected using wound-scratch and transwell migration assays. Cells (1 × 10^5^ cells per well) were seeded in triplicate into 6-well plates and cultured for 24–48 h until they reached an appropriate level of confluence. The wound-scratch assay was performed by uniformly scratching the monolayer of cells with sterile pipette tips, and the cells were washed three times with PBS. Then, serum-free medium was added in place of complete medium. Cell migration was photographed and measured in several pre-marked areas at 0 h, 24 h, and 48 h. Here, the cell migration area was measured between dashed regions by Image-J software and normalized to the control cells. The wound-healing index was calculated using the following formula: [(the wound area at 0 h) – (the wound area at indicated time)]/(the wound area at 0 h).

The transwell migration assay was performed using cell culture inserts (8 μm; BD-Falcon, Franklin Lakes, NJ, USA) inserted into a 24-well plate. Cells (5 × 10^4^ cells per insert) in 100 μL fresh medium with 1 % FBS were added to the upper chamber, while growth medium with 20 % FBS was added to the lower chamber. Plates were then incubated for 20–24 h at 37 °C. After removing the non-migrated cells and medium in the upper chamber, the migrated cells retained on the lower side of the membranes were fixed with 95 % methanol for 10 min and stained with 0.1 % crystal violet for 30 min. Photos were obtained at × 100 magnification, and cells were counted in at least three different areas. Three independent experiments were performed.

### Cell invasion assay

Invasion by control and CoCl_2_-treated MCF-7 and MDA-MB-231 cells was measured using transwell invasion assays. Cells (5 × 10^5^ per well) were seeded in 100 μL medium with 1 % FBS onto inserts pre-coated with BD Matrigel Basement Membrane Matrix (BD Biosciences). Growth medium, with 20 % FBS as the chemoattractant, was added to the bottom chamber, and plates were incubated for 24–36 h at 37 °C. After the medium and non-invaded cells in the upper chamber were removed, the transwell inserts were fixed with 95 % methanol for 10 min and stained with 0.1 % crystal violet for 30 min. Photos were taken at × 100 magnification, and the number of invaded cells was counted in multiple different fields. Three independent experiments were performed.

### Tissue samples

Paraffin-embedded human breast tumor tissue samples (n = 167) were obtained from The First Affiliated Hospital of Anhui Medical University (Hefei, China) between 2011 and 2012. None of the patients had received treatment before surgical removal of the tumor. Two pathologists verified the diagnoses of breast tumors. These 167 cases of breast tumors were divided into four groups: 52 cases of primary breast cancer with lymph node metastasis (group I), and their corresponding metastatic lymph nodes (group II); 52 cases of primary breast cancer without metastasis (group III); and 11 cases of benign breast tumors, including breast fibroadenoma and mammary gland disease (group IV). The detail information of patients in group I and group III including the ER/PR expression, lymph node metastasis and Ki-67 expression lists in Additional file 1: Table S1. The agreement for use of these tissue samples was approved by the Biomedical Ethics Committee of Anhui Medical University, and the confidentiality of patient information was maintained.

### PGCC definition and counting

PGCC are defined as cancer cells with a nucleus that is at least three times larger than that of a diploid cancer cell, as described by Zhang et al. [3]. PGCCs are not always evenly distributed throughout the sections, and the number of PGCCs in the hot spots was counted. For each case, five microscopic hot fields were counted at × 400 magnification, and the average number was calculated.

### Feulgen staining and ploidy verification

First, 4-μm sections were deparaffinized and dehydrated with xylene and ethanol. Second, the sections were hydrolyzed with 5 M HCl for 50 min at room temperature (setting for 25 – 30 °C). Third, sections were washed with 1 M HCl followed by distilled water. Finally, sections were stained in Schiff’s reagent for 60 min at room temperature, which was followed by dehydration of the sections with ethanol and xylene, and mounting with a resinous membrane.

For ploidy verification of PGCC, we used the imaging analysis software (Image-Pro Plus, Version 6.0.0.260; Media Cybernetics, Inc., Rockville, MD, USA). After Feulgen staining, typical images under the same conditions of PGCCs and control breast epithelial cells were captured. After viewing the image and choosing the area of control breast epithelial cell distribution, the software was used to calculate the area and integrated optical density (IOD). Then, we computed the mean optical density (MOD) of control breast epithelial cells. Similarly, we used the software to determine the MOD of PGCCs, and then we compared the ratio of MOD of PGCCs to that of control breast epithelial cells.

### Immunohistochemical (IHC) staining

All 4-μm-thick sections were subjected to two-step immunohistochemical (IHC) staining. Briefly, the sections were deparaffinized in xylene, and dehydrated in a set of graded ethanol solutions. Then, antigen retrieval was carried out by heating in 0.01 M citrate buffer solution (pH 6.0) with an autoclave at 120 °C for 5 min [22]. After endogenous peroxidase activity was blocked, the slides were incubated with the primary antigens overnight at 4 °C, including mouse anti-E-cadherin monoclonal antibody (MAB-0589; Maixin Bio, Fuzhou, China, 1:200 dilution), mouse anti-N-cadherin monoclonal antibody (ZM-0094; Zhongshan Inc., China, 1:100 dilution), and mouse anti-pig vimentin monoclonal antibody (kit-0019, ZM-0094; Maixin Bio, Fuzhou, China, 1:200 dilution), Slides were then washed three times with PBS for 5 min. Next, the sections were incubated with horseradish peroxidase-conjugated secondary antibody, rinsed another three times with PBS for 5 min, incubated with 3, 3'-diaminobenzidine (DAB, Zhongshan Inc.) for 1–3 min, and finally counterstained with hematoxylin. Known positive samples were used as positive controls, while the negative controls were run simultaneously by replacing the primary antibody with PBS.

### IHC scoring and quantification

Slide assessment was independently carried out by two experienced pathologists. In the evaluation of E-cadherin, N-cadherin, and vimentin expression, brown-yellow staining in the cytomembrane and/or cytoplasm was considered positive for expression. Expression within the tissue sections was quantified according to the staining intensity and percentage of positive cells. The staining intensity was scored as follows: 0, negative (no staining); 1, weak positive (faint yellow staining); 2, moderate positive (brownish-yellow staining); and 3, strong positive (brown staining). The number of positive cells was visually evaluated and stratified as follows: 0 (negative), <5 % positive cells; 1 (weak), 6 %-25 % positive cells; 2 (moderate), 26 %-50 % positive cells; 3 (above moderate), 51 %-75 %; and 4 (strong), >76 % positive cells [21]. The sum of the staining intensity and positive cell scores was used to determine the staining index for each section [23].

### Statistical analysis

Statistical software SPSS 17.0 for Windows was used to analyze all the statistical data in this study. All histogram data are presented as mean ± SD, and all table data are presented as mean ± SEM. The Kruskal-Wallis test was performed to compare the differences in PGCC number and EMT-related protein expression among the four groups. The Mann–Whitney test was used to analyze the differences in PGCC number and EMT-related protein expression between two groups of the four. Other comparisons were performed with a two-tailed Student’s *t*-test. Here, a *p*-value < 0.05 was considered statistically significant.

## Results

### CoCl_2_-induced PGCC formation in breast cancer cells

**Fig. 1:**
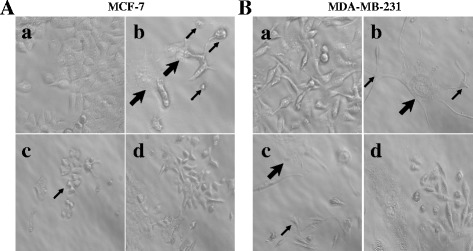
PGCCs with budding daughter cells in MCF-7 and MDA-MB-231 cells. **A**. MCF-7 PGCCs and control MCF-7 cells. a. Control MCF-7 cells (×400). b. MCF-7 PGCCs induced by 450 μM CoCl_2_ treatment for 72 h (×400). Small black arrowheads indicate budded daughter cells; large black arrow heads indicate PGCCs. c. PGCCs generated daughter cells via budding 10–15 days after CoCl_2_ treatment. Black arrowheads indicate budded daughter cells (×100). d. Fast reproduction of PGCCs by generated daughter cells via budding (×100). **B**. MDA-MB-231 PGCCs and control MDA-MB-231 cells. a. Control MDA-MB-231 cells (×400). b. MDA-MB-231 PGCCs induced by 300 μM CoCl_2_ treatment for 72 h (×400). Small black arrowheads indicate budded daughter cells; large black arrowheads indicate PGCCs. c. PGCCs generated daughter cells via budding 10–15 days after CoCl_2_ treatment. Small black arrowheads indicate budded daughter cells; large black arrowheads indicate PGCCs (×100). d. Recovery of PGCCs by generated budding daughter cells (×100)

When the breast cancer cell lines MCF-7 and MDA-MB-231 were treated with high concentrations of CoCl_2_ over a long period, normal-sized diploid cancer cells were selectively killed while some large cells with giant nuclei (PGCCs) survived. After a high concentration CoCl_2_ treatment (450 μM) of MCF-7 cells for 72 h killed most diploid cells, PGCCs could be observed after removal of floating dead cells, in contrast to the control MCF-7 cells (Fig. 1A-a and A-b). Similar morphological changes were observed for the MDA-MB-231 breast cancer cell line (Fig. 1B-a and B-b). At 10–15 days post-CoCl_2_ treatment, the surviving PGCCs cultured in complete medium generated daughter cells via budding (Fig. 1A-c and B-c). The number of regular-sized daughter cells increased from 60 % to 70 % after 24 h of continuous culture in complete medium, while the percentage of PGCCs in the flask decreased from 40 % to 30 % (Fig. 1A-d and B-d). These PGCCs with budding cells and control cells were used for the cell cycle and EMT-related protein expression analyses.

### Cell cycle analysis in breast cancer cells before and after CoCl_2_ treatment

**Fig. 2:**
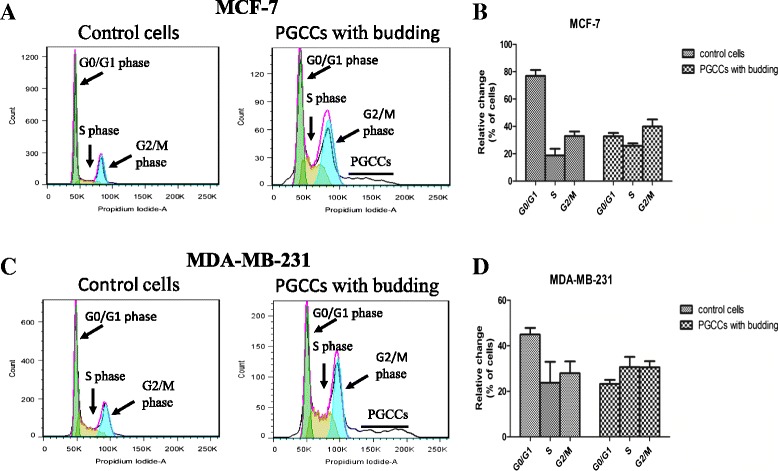
Alteration of cell cycle in breast cancer cell lines after CoCl_2_ treatment. Clear alteration of the cell cycle as analyzed by flow cytometry was observed in MCF-7 (**a**) and MDA-MB-231 (**c**) cells treated with CoCl_2_. The bar graphs in (**b**) and (**d**) depict the relative changes to cell percentage in different phases of the cell cycle in PGCCs with budding compared to that of control cells. All data represent the mean ± SD of three independent cultures

CoCl_2_-induced alterations to the cell cycle in various phases in MCF-7 and MDA-MB-231 cells was observed using flow cytometry (Fig. 2a and c). We observed a sharp decrease in the number of CoCl_2_-treated cells in G1 phase, but an increase in the number of cells in S and G2 phases (Fig. 2b and d). In addition, flow cytometric analysis confirmed that cells treated with CoCl_2_ generated a cell subpopulation corresponding to PGCCs, which had a large increase in DNA copy number.

### EMT-related protein expression in control breast cancer cells and PGCCs with budding

**Fig. 3:**
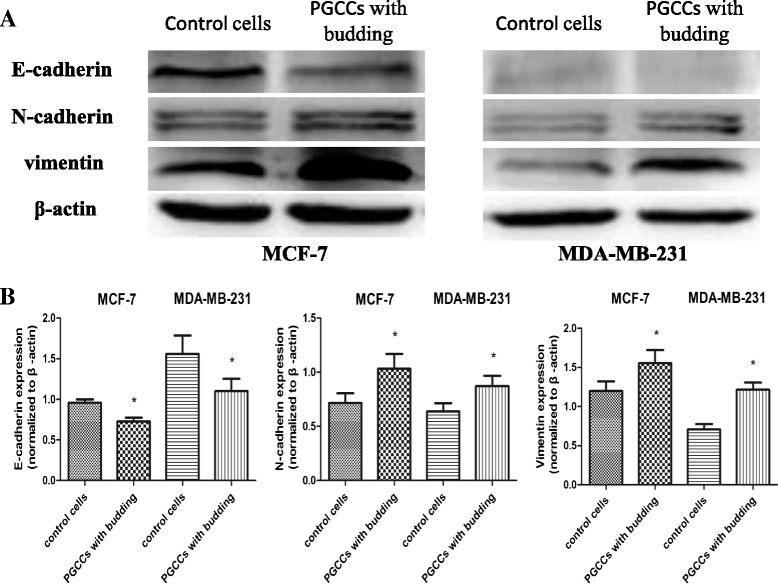
E-cadherin, N-cadherin, and vimentin expression in PGCCs with budding and control MCF-7 and MDA-MB-231 cells. **a** Western blot was used to detect differences in E-cadherin, N-cadherin, and vimentin expression in MCF-7 and MDA-MB-231 cells before and after CoCl_2_ treatment. **b** Quantitative results of protein expression differences are shown as histograms. The corresponding densitometric analyses of the protein bands performed using Image-J software were normalized to the signal of β-actin. Each bar represents the mean ± SD of three independent experiments (*p < 0.05)

The expression of EMT-related proteins, including E-cadherin, N-cadherin, and vimentin, was analyzed in control MCF-7 and MDA-MB-231 cells and in PGCCs with budding cells. Western blot analysis revealed that E-cadherin expression was lower in PGCCs with budding than in control cells, and that expression of N-cadherin and vimentin was greater in PGCCs with budding than in control cells (Fig. 3a). A quantitative analysis of EMT-related protein expression in PGCCs with budding and control cells is depicted in Fig. 3b. The densitometric analyses of each protein band were normalized to the corresponding β-actin band, which revealed a significant difference between PGCCs with budding and control cells.

### Daughter cells budded by PGCCs have great migration and invasion abilities

**Fig. 4:**
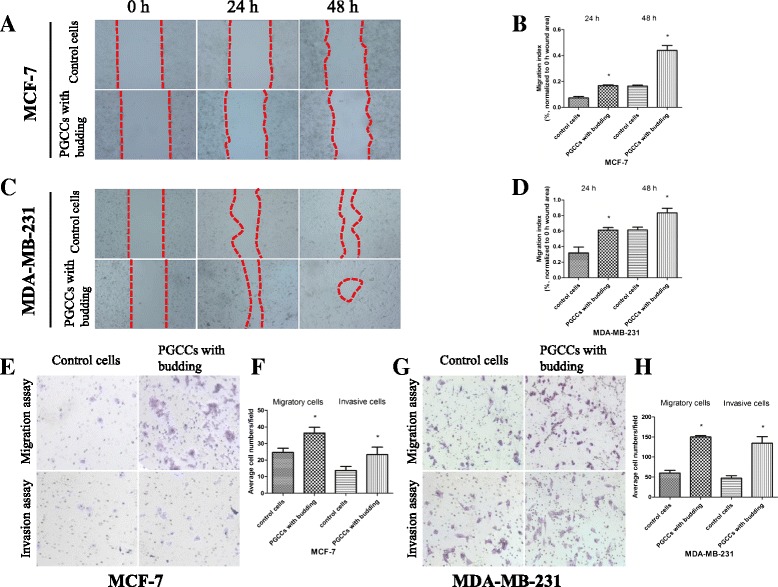
CoCl_2_ increases the migration and invasion of breast cancer cells. **a** Representative images of the wound-healing assay for MCF-7 cells at different times (×40). **b** MCF-7 cell migration is shown as a wound-healing index quantified by measuring at least three different wound areas. **c** Representative images of the wound-healing assay for MDA-MB-231 cells at different times (×40). **d** Quantitative data of MDA-MB-231 cell migration between control cells and PGCCs with budding. **e**, **g** Transwell migration and invasion assays were performed in control MCF-7 and MDA-MB-231 cells and PGCCs with budding (×100). Upper panels indicate the migration and lower panels show cell invasion. **f**, **h** Quantitative results of transwell migration and invasion assay in MCF-7 and MDA-MB-231 cells

To determine whether CoCl_2_ treatment affects cell migration in the breast cancer cell lines MCF-7 and MDA-MB-231, a wound-healing assay was performed on PGCCs with budding and control cells. Fig. 4a and c depict the wound-scratch assay at 0 h, 24 h, and 48 h. The spaces between the red dashed lines gradually narrowed in the panels. The quantitative analysis reveals a significant increase in the cell migration ability of PGCCs with budding (Fig. 4b and d). Moreover, we also performed a transwell migration assay with MCF-7 and MDA-MB-231 cells to compare PGCCs with budding and control cells. As shown in Fig. 4e and g, the number of migrated cells increased in PGCCs with budding cells compared with the control cells. To examine whether CoCl_2_ treatment affects cell invasion in MCF-7 and MDA-MB-231 cells, the cell invasion assay was performed using matrigel-coated transwell inserts. Invaded cell numbers also greatly increased in the group of PGCCs with budding cells compared to control cells (Fig. 4e and g). Quantitative results of transwell migration and invasion assays in MCF-7 and MDA-MB-231 cells indicated significant differences in the numbers of migratory and invasive cells (Fig. 4f and h).

### Clinicopathological significance of the number of PGCCs in human breast tumors

**Fig. 5:**
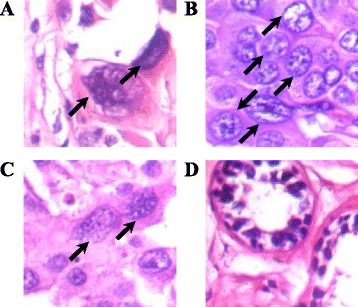
PGCCs in human breast tumors. **a** PGCCs in primary breast cancer with metastasis (group I; black arrowheads, H&E, ×400). **b** PGCCs in metastatic tumors (group II; black arrowheads, H&E, ×400). **c** PGCCs in primary breast cancer without metastasis (group III; black arrowheads, H&E, ×400). **d** PGCCs are absent in benign breast tumors (group IV; H&E, ×400)

Using the description of PGCCs set by Zhang et al. [3], we observed morphologically that PGCCs with giant or multiple nuclei were significantly present in human breast tumors of group I, group II, and group III (Fig. 5). As shown in Table 1, group II had the highest number of PGCCs and group III had the lowest; the difference between these groups was statistically significant (*P* = 0.000). PGCCs were not observed in group IV. The average number of PGCCs was higher in group II than in group I (Z = −7.402, *P* = 0.000), higher in group I than in group III (Z = −6.278, *P* = 0.000), and higher in group III than in group IV (Z = −5.196, *P* = 0.000).

**Table 1:** Comparison of the average number of PGCCs in human breast tumors

	Group	Number	Number of PGCCs	χ^2^	*P* value
Primary breast cancer with lymph node metastasis	I	52	22.62 ± 1.15	125.193	0.000
Corresponding metastatic tumor	II	52	41.04 ± 1.49		
Primary breast cancer without metastasis	III	52	11.35 ± 0.90		
Benign breast tumor	IV	11	0		

### Feulgen staining and mean optical density determination

**Fig. 6:**
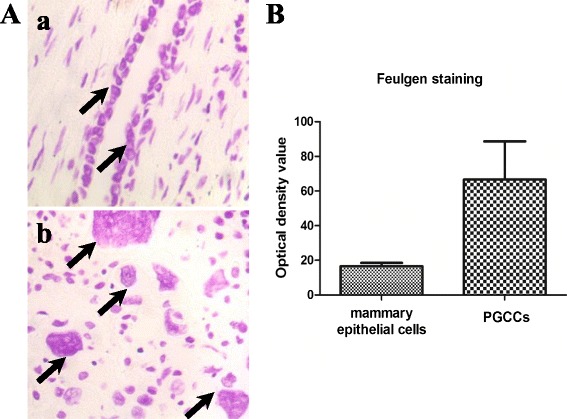
Feulgen staining and MOD determination of PGCCs and control mammary epithelial cells. **A**. Feulgen staining between PGCCs and control mammary epithelial cells. a. Feulgen staining of mammary epithelial cells (*black arrowheads*, ×200). b. Feulgen staining of PGCCs in breast cancer (*black arrowheads*, ×200). **B**. Quantitative results of MOD determination in PGCCs and control mammary epithelial cells

To observe the nuclei morphologies of PGCCs in human breast tumor tissues, we performed Feulgen staining. Feulgen staining, in which DNA is dyed purple, revealed the distribution of DNA in tumor cells (Fig. 6a). The images were captured at × 200 magnification, analyzed by Image-Pro Plus software, and the MOD of control breast epithelial cells and PGCCs was calculated. Three further images of normal breast tissue and breast cancer were obtained and analyzed. By analyzing these images, we determined an average optical density value for normal breast epithelium, fluctuating between 14 and 19, and determined the MOD of PGCCs to be in the range of 40 to 90, positively correlating with the volume of PGCCs. The ratio of PGCC and control breast epithelial cell approximations was an integer greater than or equal to 2. Compared with normal diploid mammary epithelial cells, the polyploidy nature of PGCCs human breast tumor tissue was confirmed. Quantitative results of MOD determinations are shown in Fig. 6b.

### Expression of EMT-related proteins in human breast tumor tissues

**Fig. 7:**
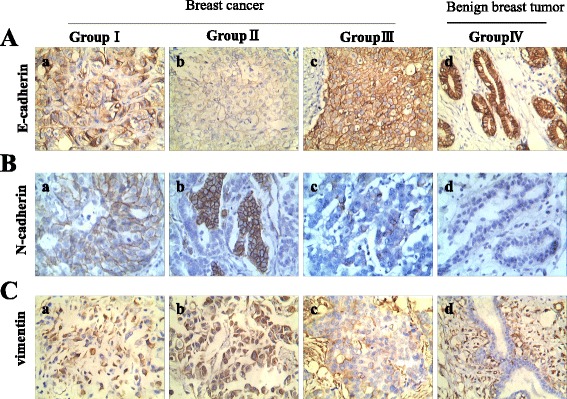
Expression of E-cadherin, N-cadherin, and vimentin in human breast tumor tissues. **A**. E-cadherin expression in (a) primary breast cancer with lymph node metastasis (group I), (b) corresponding metastatic cancer (group II), (c) primary breast cancer without metastasis (group III), and (d) benign breast tumor (group IV) (×200). **B**. N-cadherin expression in (a) primary breast cancer with lymph node metastasis (group I), (b) corresponding metastatic tumor (group II), (c) primary breast cancer without metastasis (group III), and (d) benign breast tumor (group IV) (×200). **C**. Vimentin expression in (a) primary breast cancer with lymph node metastasis (group I), (b) corresponding metastatic tumor (group II), (c) primary breast cancer without metastasis (group III), and (d) benign breast tumor (group IV) (×200)

**Fig. 8:**
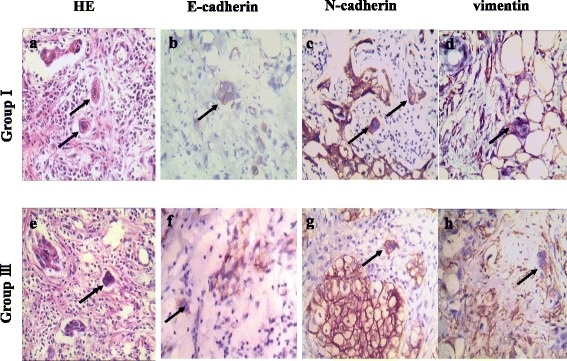
Expression of E-cadherin, N-cadherin, and vimentin in single stroma PGCCs of human breast cancer. **a** Single stroma PGCC in human breast cancer with lymph node metastasis (*Black arrow head*, H&E, ×200). **b** E-cadherin, (**c**) N-cadherin, (**d**) vimentin expression of single stroma PGCC in human breast cancer with lymph node metastasis (*Black arrow head*, IHC,×200). **e** Single stroma PGCC in human breast cancer without metastasis (*Black arrow head*, H&E, ×200). **b** E-cadherin, (**c**) N-cadherin, (**d**) vimentin expression of single stroma PGCC in human breast cancer without metastasis (*Black arrow head*, IHC, ×200)

To detect the expression level of EMT-related proteins and their clinicopathological significance, IHC staining for E-cadherin, N-cadherin, and vimentin was performed on 167 cases of formalin-fixed, paraffin-embedded human breast tumor tissues. Positive E-cadherin (Fig. 7a) staining appeared in the cytomembrane or cytoplasm of tumor cells, positive N-cadherin (Fig. 7b) staining was detected in the cytomembrane, and positive vimentin staining was present in the cytoplasm (Fig. 7c). E-cadherin (*P* = 0.000), N-cadherin (*P* = 0.000), and vimentin (*P* = 0.000) staining indexes were significantly different between the four groups (Table 2). Metastatic cancer (group II) exhibited the highest N-cadherin and vimentin expression, and the lowest E-cadherin expression (Fig. 7 and Table 2). Benign breast tumors (group IV) had the highest E-cadherin expression, and the lowest N-cadherin and vimentin expression (Fig. 7 and Table 2). Statistical analysis revealed that the expression of N-cadherin and vimentin was higher in group II than in group I (Z = −2.856, *P* = 0.004; Z = −2.347, *P* = 0.019; respectively; Table 3), higher in group I than in group III (Z = −2.736, *P* = 0.006; Z = −3.545, *P* = 0.000; respectively; Table 4), and higher in group III than in group IV (Z = −1.592, *P* = 0.111; Z = −2.524, *P* = 0.012; respectively; Table 5). The staining indices of N-cadherin and vimentin were significantly different between group I and group II and between group I and group III. Moreover, the expression of E-cadherin was lower in group II than in group I (Z = −2.713, *P* = 0.007; Table 3), lower in group I than in group III (Z = −2.720, *P* = 0.007; Table 4), and lower in group III than in group IV (Z = −1.246, *P* = 0.213; Table 5). The differences in E-cadherin expression were statistically significant between group I and group II, and between group I and group III. Furthermore, PGCCs in group I (Fig. 8 –a) and group III (Fig. 8 -e) undergo EMT and IHC staining for E-cadherin, N-cadherin and vimentin in group I (Fig. 8 –b,-c,-d) and group III (Fig. 8 –f,-g,-h) were performed. Results of IHC staining showed that single stroma PGCC located in the invasive front of primary breast cancer with metastasis were strong positive for N-cadherin (Fig. 8 -c) and vimentin (Fig. 8 -d) and weak positive for E-cadherin (Fig. 8 -b). Single stroma PGCC in group III had the similar expression of N-cadherin (Fig. 8 -g), vimentin (Fig. 8 -h) and E-cadherin (Fig. 8 -e) as it in primary breast cancer with metastasis.

**Table 2:** The differences of E-cadherin, N-cadherin, and vimentin expression in human breast tumors

	Group	Number	E-cadherin	N-cadherin	vimentin
Primary breast tumor with metastasis	I	52	9.67 ± 0.34	0.96 ± 0.19	1.96 ± 0.24
Corresponding metastatic tumor	II	52	8.38 ± 0.34	1.81 ± 0.28	3.02 ± 0.35
Primary breast tumor without metastasis	III	52	10.90 ± 0.26	0.46 ± 0.12	0.92 ± 0.15
Benign breast tumor	IV	11	11.64 ± 0.36	0.09 ± 0.09	0.18 ± 0.12
χ^2^			35.267	31.455	45.777
*P*			0.000	0.000	0.000

**Table 3:** The differences of E-cadherin, N-cadherin, and vimentin expression between group I and group II

	Group	Number	E-cadherin	N-cadherin	vimentin
Primary breast tumor with metastasis	I	52	9.67 ± 0.34	0.96 ± 0.19	1.96 ± 0.24
Corresponding metastatic tumor	II	52	8.38 ± 0.34	1.81 ± 0.28	3.02 ± 0.35
Z			−2.713	−2.856	−2.347
*P*			0.007	0.004	0.019

**Table 4:** The differences of E-cadherin, N-cadherin, and vimentin expression between group I and group III

	Group	Number	E-cadherin	N-cadherin	vimentin
Primary breast tumor with metastasis	I	52	9.67 ± 0.34	0.96 ± 0.19	1.96 ± 0.24
Primary breast tumor without metastasis	III	52	10.90 ± 0.26	0.46 ± 0.12	0.92 ± 0.15
Z			−2.720	−2.736	−3.545
*P*			0.007	0.006	0.000

**Table 5:** The differences of E-cadherin, N-cadherin, and vimentin expression between group III and group IV

	Group	Number	E-cadherin	N-cadherin	vimentin
Primary breast tumor without metastasis	III	52	10.90 ± 0.26	0.46 ± 0.12	0.92 ± 0.15
Benign breast tumor	IV	11	11.64 ± 0.36	0.09 ± 0.09	0.18 ± 0.12
Z			−1.246	−1.592	−2.524
*P*			0.213	0.111	0.012

## Discussion

PGCCs are large and contain single giant nuclei or multiple nuclei and are a special sub-population of cancer cells that contribute to solid tumor heterogeneity. PGCCs are the most commonly described histopathologic features of human tumors, particularly in high-grade and advanced-stage tumors, and usually correlate with poor prognosis (ref) (Malpica et al., 2004; Polyak, 2011; Wolberg et al., 1999). PGCCs have been considered as senescent cells, or as at the stage of mitotic catastrophe, but our previous data revealed that these large cancer cells were actually live and could generate daughter cancer cells via budding [3, 4]. PGCCs were initially observed in flasks after high concentrations of CoCl_2_ treatment, which selectively killed regular diploid cells of HEY, SKOV3, and MDA-MB-231 cancer cells [3]. CoCl_2_ can induce the formation of PGCCs through endoreduplication or cell fusion, and PGCCs can generate daughter cancer cells via asymmetric cell division, including splitting, budding, and burst, which commonly appears in lower organisms [3, 9, 10]. The daughter cells generated by PGCCs exhibit mesenchymal phenotypes, and have stronger migration and invasion abilities than those of regular diploid cancer cells. In this paper, we characterized a PGCC as a cancer cell with a nucleus at least three times larger than that of a diploid cancer cell.

Recently, more studies have confirmed that EMT plays an important role in tumor invasion and metastasis [24–26]. In the present study, we investigated the expression of three EMT-related proteins, including E-cadherin, N-cadherin, and vimentin, in PGCCs with budding daughter cells and control cancer cells without CoCl_2_ treatment. Furthermore, the expression of these proteins was compared in human breast tumor tissues, and IHC staining showed that expression of these proteins was associated with breast tumor lymph node metastasis.

E-cadherin, a calcium-dependent single-span transmembrane glycoprotein, has been widely known as a tumor suppressor and a characterized molecular marker of EMT [27], and is an important epithelial cell adhesion molecule and signal transduction factor located in the epithelial tissue [25]. The cytoplasmic domain of E-cadherin interacts with many proteins, including three catenins (α, β, and p120), and combines with the actin cytoskeleton. E-cadherin sets up close alloantigen interactions with contiguous E-cadherin molecules in neighboring cells to form the core of the epithelial adherens junction [28, 29]. Loss of E-cadherin can lead to enhanced tumor mobility and invasion [30, 31].

N-cadherin is also a calcium-dependent glycoprotein. Unlike E-cadherin, N-cadherin is absent in normal epithelium, and promotes a dynamic adhesion state in tumor cells, allowing not only the separation of single cells from the tumor entity but also their interactions with endothelial and stromal components [32–34]. Expression of N-cadherin is further increased in tumors with metastatic potential and is up-regulated in many cancers, including breast invasive ductal carcinomas [24, 34, 35], colorectal cancers [36, 37], esophageal squamous cell carcinomas [21], and non-small cell lung cancers [38].

Vimentin, an important component of the cytoskeleton, is widely distributed in mesenchymal cells such as endothelial cells, lymphocytes, and fibroblasts [25, 39]. As another well-known marker of EMT, several studies have shown that the abnormal expression of vimentin was present in many kinds of epithelial tumors and associated with the invasion and metastasis of cancer cells [40, 41]. Furthermore, the overexpression of vimentin in breast cancer cells is correlated with poor prognosis, leading to adverse clinicopathological features in patients [39, 42]. EMT is known as a cadherin switching process, leading to primary molecular changes, including the loss of epithelial cell markers and overexpression of mesenchymal cell markers [43–45].

In the present study, we confirmed that the expression of E-cadherin was down-regulated, and that the expression of N-cadherin and vimentin was up-regulated in PGCCs with budding daughter cells. By detecting EMT-related proteins in cancer cell lines, we estimate that the migration and invasion ability of the daughter cells budded from PGCCs could be attributed to EMT. Furthermore, more PGCCs were detected in the group of breast tumors with metastasis than in the group without metastasis. Finally, expression of E-cadherin was down-regulated, while expression of N-cadherin and vimentin was up-regulated, in primary breast carcinomas with metastasis and their metastatic foci, compared with primary breast carcinomas without metastasis and benign breast lesions. These data further demonstrate that the number of PGCCs and EMT-related protein expression are associated with invasion and metastasis in breast cancer. However, the mechanisms of PGCCs formation and EMT involving in daughter cells generated by PGCCs via budding are still unclear. Sequencing and cGH analysis focused on the genetic heterogeneity of PGCCs at the levels of mutation and genomic rearrangements need to be performed in the future.

## Conclusions

The current study introduces the novel concept that PGCCs are present in human breast cancer, and that PGCCs with their newly budding daughter cells contribute to the occurrence of EMT-associated cancer invasion and metastasis. Further exploration of the mechanisms of PGCC formation and EMT-related protein expression in the development, invasion, and metastasis of breast cancer is needed.

## Additional file

Additional file 1: Table-S1. Detail information of patients in group I and group III. (DOC 27 kb)

## Abbreviations

CoCl_2_: cobalt chloride
DAB: diaminobenzidine
EMT: epithelial-mesenchymal transition
FBS: fetal bovine serum
FISH: fluorescence in situ hybridization
IHC: immunohistochemistry
IOD: integrated optical density
MOD: mean optical density
PBS: phosphate-buffered saline
PGCCs: Polyploid giant cancer cells
PVDF Membrane: polyvinylidene fluoride membrane
SDS: sodium dodecyl sulfate

## References

[1] Torre LA, Bray F, Siegel RL, Ferlay J, Lortet-Tieulent J, Jemal A (2015). Global cancer statistics, 2012. CA Cancer J Clin.

[2] Kennecke H, Yerushalmi R, Woods R, Cheang MC, Voduc D, Speers CH (2010). Metastatic behavior of breast cancer subtypes. J. Clin. Oncol. Off. J. Am. Soc. Clin. Oncol..

[3] Zhang S, Mercado-Uribe I, Xing Z, Sun B, Kuang J, Liu J (2014). Generation of cancer stem-like cells through the formation of polyploid giant cancer cells. Oncogene.

[4] Zhang S, Mercado-Uribe I, Liu J (2013). Generation of erythroid cells from fibroblasts and cancer cells in vitro and in vivo. Cancer Lett.

[5] Marusyk A, Almendro V, Polyak K (2012). Intra-tumour heterogeneity: a looking glass for cancer?. Nat Rev Cancer.

[6] Zhang S, Zhang D, Zhu Y, Guo H, Zhao XBS (2006). Clusterin expression and univariate analysis of overall survival in human breast cancer. Technol Cancer Res Treat.

[7] Lopez-Sánchez LM JC, Valverde A, Hernandez V, Peñarando J, Martinez A, Lopez-Pedrera C (2014). CoCl2, a Mimic of Hypoxia, Induces Formation of Polyploid Giant Cells with Stem Characteristics in Colon Cancer. PLoS One.

[8] Zhang D, Wang Y, Zhang S (2014). Asymmetric cell division in polyploid giant cancer cells and low eukaryotic cells. BioMed Res Int.

[9] Zhang S, Mercado-Uribe I, Liu J (2014). Tumor stroma and differentiated cancer cells can be originated directly from polyploid giant cancer cells induced by paclitaxel. Int. J. Cancer: Journal international du cancer.

[10] Zhang S, Mercado-Uribe I, Hanash S, Liu J (2013). iTRAQ-based proteomic analysis of polyploid giant cancer cells and budding progeny cells reveals several distinct pathways for ovarian cancer development. PLoS One.

[11] Vitale I, Senovilla L, Jemaa M, Michaud M, Galluzzi L, Kepp O (2010). Multipolar mitosis of tetraploid cells: inhibition by p53 and dependency on Mos. EMBO J.

[12] Erenpreisa J, Salmina K, Huna A, Kosmacek EA, Cragg MS, Ianzini F (2011). Polyploid tumour cells elicit paradiploid progeny through depolyploidizing divisions and regulated autophagic degradation. Cell Biol Int.

[13] Mani SA, Guo W, Liao MJ, Eaton EN, Ayyanan A, Zhou AY (2008). The epithelial-mesenchymal transition generates cells with properties of stem cells. Cell.

[14] Greenburg G, Hay ED (1982). Epithelia suspended in collagen gels can lose polarity and express characteristics of migrating mesenchymal cells. J Cell Biol.

[15] Zhao Z, Lu P, Zhang H, Xu H, Gao N, Li M (2014). Nestin positively regulates the Wnt/β-catenin pathway and the proliferation, survival and invasiveness of breast cancer stem cells. Breast Cancer Res.

[16] Tran DD, Corsa CA, Biswas H, Aft RL, Longmore GD (2011). Temporal and spatial cooperation of Snail1 and Twist1 during epithelial-mesenchymal transition predicts for human breast cancer recurrence. Mol. Cancer Res.: MCR.

[17] Jin HMS, Sato F, Kudo Y, Akasaka H, Tsutsumi S, Ogasawara H (2010). Vimentin expression of esophageal squamous cell carcinoma and its aggressive potential for lymph node metastasis. Biomed Res.

[18] Li K, Wang X, He W, Lin N, Fan QX (2009). Expression of N-cadherin in esophageal squamous cell carcinoma and silencing expression of N-cadherin using RNA interference on invasiveness of EC9706 cells. Ai Zheng.

[19] Onder TT, Gupta PB, Mani SA, Yang J, Lander ES, Weinberg RA (2008). Loss of E-cadherin promotes metastasis via multiple downstream transcriptional pathways. Cancer Res.

[20] Kumar KJ, Vani MG, Chueh PJ, Mau JL, Wang SY (2015). Antrodin C inhibits epithelial-to-mesenchymal transition and metastasis of breast cancer cells via suppression of Smad2/3 and beta-catenin signaling pathways. PLoS One.

[21] Pang L, Li Q, Wei C, Zou H, Li S, Cao W (2014). TGF-beta1/Smad signaling pathway regulates epithelial-to-mesenchymal transition in esophageal squamous cell carcinoma: in vitro and clinical analyses of cell lines and nomadic Kazakh patients from northwest Xinjiang, China. PLoS One.

[22] Li KDH, Lian X, Yang S, Chai D, Wang C, Yang R (2014). Characterization of β2-microglobulin expression in different types of breast cancer. BMC Cancer.

[23] Di Martino E, Wild CP, Rotimi O, Darnton JS, Olliver RJ, Hardie LJ (2006). IGFBP-3 and IGFBP-10 (CYR61) up-regulation during the development of Barrett's oesophagus and associated oesophageal adenocarcinoma: potential biomarkers of disease risk. Biomarkers: Biochemical indicators of exposure, response, and susceptibility to chemicals.

[24] ElMoneim HM, Zaghloul NM (2011). Expression of e-cadherin, n-cadherin and snail and their correlation with clinicopathological variants: an immunohistochemical study of 132 invasive ductal breast carcinomas in Egypt. Clinics (Sao Paulo).

[25] Zhou J, Tao D, Xu Q, Gao Z, Tang D (2015). Expression of E-cadherin and vimentin in oral squamous cell carcinoma. Int J Clin Exp Pathol.

[26] Qu BL, Yu W, Huang YR, Cai BN, Du LH, Liu F (2015). 6-OH-BDE-47 promotes human lung cancer cells epithelial mesenchymal transition via the AKT/Snail signal pathway. Environ Toxicol Pharmacol.

[27] Le Bras GF, Taubenslag KJ, Andl CD (2012). The regulation of cell-cell adhesion during epithelial-mesenchymal transition, motility and tumor progression. Cell Adhes Migr.

[28] Gumbiner BM (2005). Regulation of cadherin-mediated adhesion in morphogenesis. Nat Rev Mol Cell Biol.

[29] Nagafuchi A, Shirayoshi Y, Okazaki K, Yasuda K, Takeichi M (1987). Transformation of cell adhesion properties by exogenously introduced E-cadherin cDNA. Nature.

[30] Derksen PW, Liu X, Saridin F, van der Gulden H, Zevenhoven J, Evers B (2006). Somatic inactivation of E-cadherin and p53 in mice leads to metastatic lobular mammary carcinoma through induction of anoikis resistance and angiogenesis. Cancer Cell.

[31] Baranwal S, Alahari SK (2009). Molecular mechanisms controlling E-cadherin expression in breast cancer. Biochem Biophys Res Commun.

[32] Tomita K, van Bokhoven A, van Leenders GJ, Ruijter ET, Jansen CF, Bussemakers MJ (2000). Cadherin switching in human prostate cancer progression. Cancer Res.

[33] Li G, Herlyn M (2000). Dynamics of intercellular communication during melanoma development. Mol Med Today.

[34] Hazan RB, Phillips GR, Qiao RF, Norton L, Aaronson SA (2000). Exogenous expression of N-cadherin in breast cancer cells induces cell migration, invasion, and metastasis. J Cell Biol.

[35] Nagi C, Guttman M, Jaffer S, Qiao R, Keren R, Triana A (2005). N-cadherin expression in breast cancer: correlation with an aggressive histologic variant--invasive micropapillary carcinoma. Breast Cancer Res Treat.

[36] Ye Z, Zhou M, Tian B, Wu B, Li J (2015). Expression of lncRNA-CCAT1, E-cadherin and N-cadherin in colorectal cancer and its clinical significance. Int J Clin Exp Med.

[37] Yan X, Yan L, Liu S, Shan Z, Tian Y, Jin Z (2015). N-cadherin, a novel prognostic biomarker, drives malignant progression of colorectal cancer. Mol. Med. Rep..

[38] Hui L, Zhang S, Dong X, Tian D, Cui Z, Qiu X (2013). Prognostic significance of twist and N-cadherin expression in NSCLC. PLoS One.

[39] Karihtala P, Auvinen P, Kauppila S, Haapasaari KM, Jukkola-Vuorinen A, Soini Y (2013). Vimentin, zeb1 and Sip1 are up-regulated in triple-negative and basal-like breast cancers: association with an aggressive tumour phenotype. Breast Cancer Res Treat.

[40] Yamashita N, Tokunaga E, Kitao H, Hisamatsu Y, Taketani K, Akiyoshi S (2013). Vimentin as a poor prognostic factor for triple-negative breast cancer. J Cancer Res Clin Oncol.

[41] Yao X, Wang X, Wang Z, Dai L, Zhang G, Yan Q (2012). Clinicopathological and prognostic significance of epithelial mesenchymal transition-related protein expression in intrahepatic cholangiocarcinoma. OncoTargets and Therapy.

[42] Liu T, Zhang X, Shang M, Zhang Y, Xia B, Niu M (2013). Dysregulated expression of Slug, vimentin, and E-cadherin correlates with poor clinical outcome in patients with basal-like breast cancer. J Surg Oncol.

[43] Wheelock MJ, Shintani Y, Maeda M, Fukumoto Y, Johnson KR (2008). Cadherin switching. J Cell Sci.

[44] Scanlon CS, Van Tubergen EA, Inglehart RC, D'Silva NJ (2013). Biomarkers of epithelial-mesenchymal transition in squamous cell carcinoma. J Dent Res.

[45] Turley EA, Veiseh M, Radisky DC, Bissell MJ (2008). Mechanisms of disease: epithelial-mesenchymal transition--does cellular plasticity fuel neoplastic progression?. Nat Clin Pract Oncol.

