# Correction: The number of polyploid giant cancer cells and epithelial-mesenchymal transitionrelated proteins are associated with invasion and metastasis in human breast cancer

**DOI:** 10.1186/s13046-024-03148-y

**Published:** 2024-08-08

**Authors:** Fei Fei, Dan Zhang, Zhengduo Yang, Shujing Wang, Xian Wang, Zhengsheng Wu, Qiang Wu, Shiwu Zhang

**Affiliations:** 1 Department of Pathology, Anhui Medical University, Hefei, Anhui 230032 People’s Republic of China; 2 Department of Pathology, Tianjin Union Medicine Center, 300121 Tianjin, People’s Republic of China

**Correction: J Exp Clin Cancer Res 34, 158 (2015)**

10.1186/s13046-015-0277-8

Following publication of the original article [1], the authors found an overlapping image in the lower left corner of “Control cells” (left image) and the upper right corner of “PGCCs with budding” (right image) in the “Invasion assay” section of Fig. 4, panel G.

Incorrect Figure 4

**Fig. 4:**
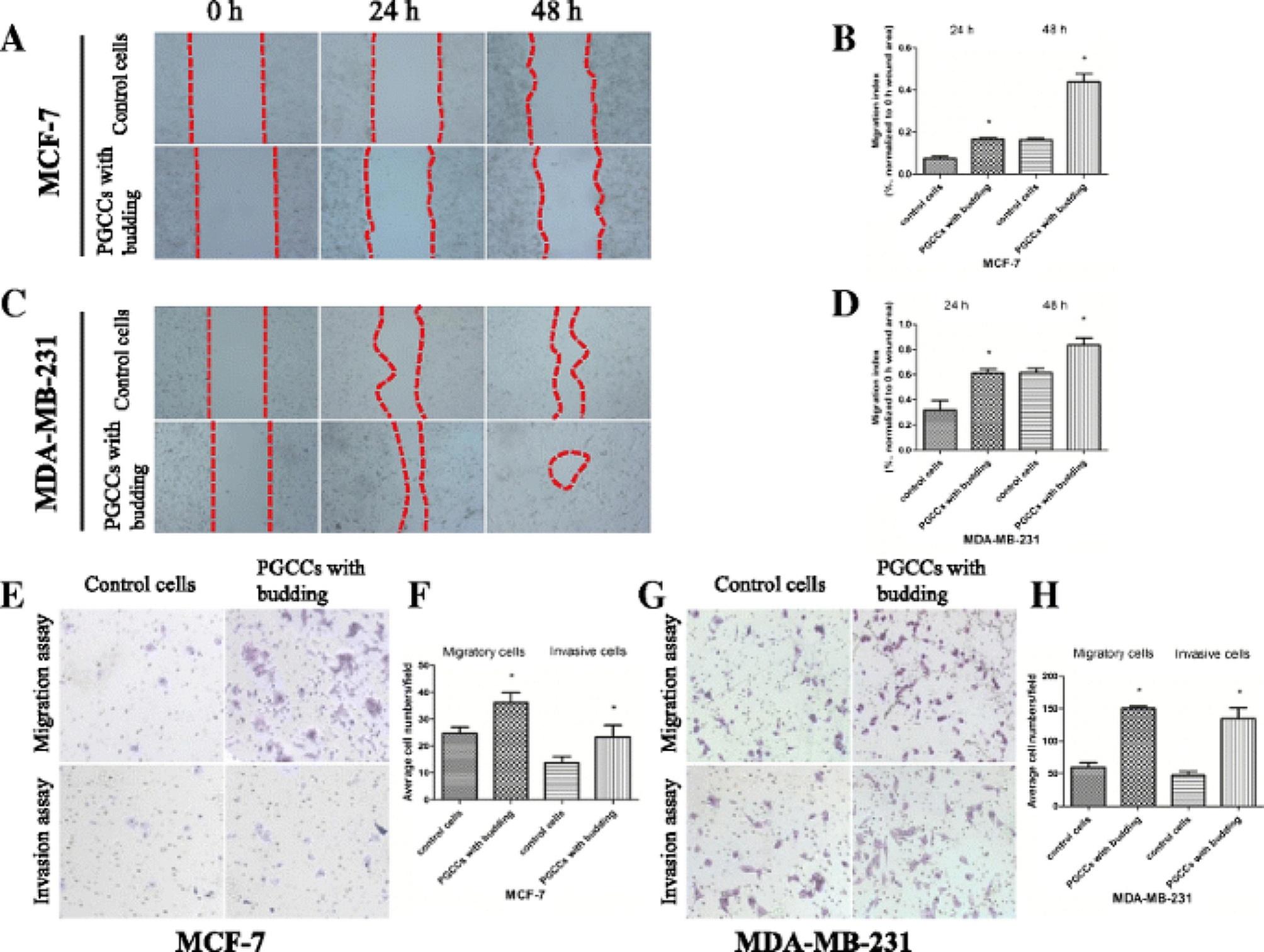
CoCl_2_ increases the migration and invasion of breast cancer cells. **a** Representative images of the wound-healing assay for MCF-7 cells at different times (×40). **b** MCF-7 cell migration is shown as a wound-healing index quantified by measuring at least three different wound areas. **c** Representative images of the wound-healing assay for MDA-MB-231 cells at different times (×40). **d** Quantitative data of MDA-MB-231 cell migration between control cells and PGCCs with budding. **e**,** g** Transwell migration and invasion assays were performed in control MCF-7 and MDA-MB-231 cells and PGCCs with budding (×100). Upper panels indicate the migration and lower panels show cell invasion. **f**,** h** Quantitative results of transwell migration and invasion assay in MCF-7 and MDA-MB-231 cells

Correct Figure 4

**Fig. 4:**
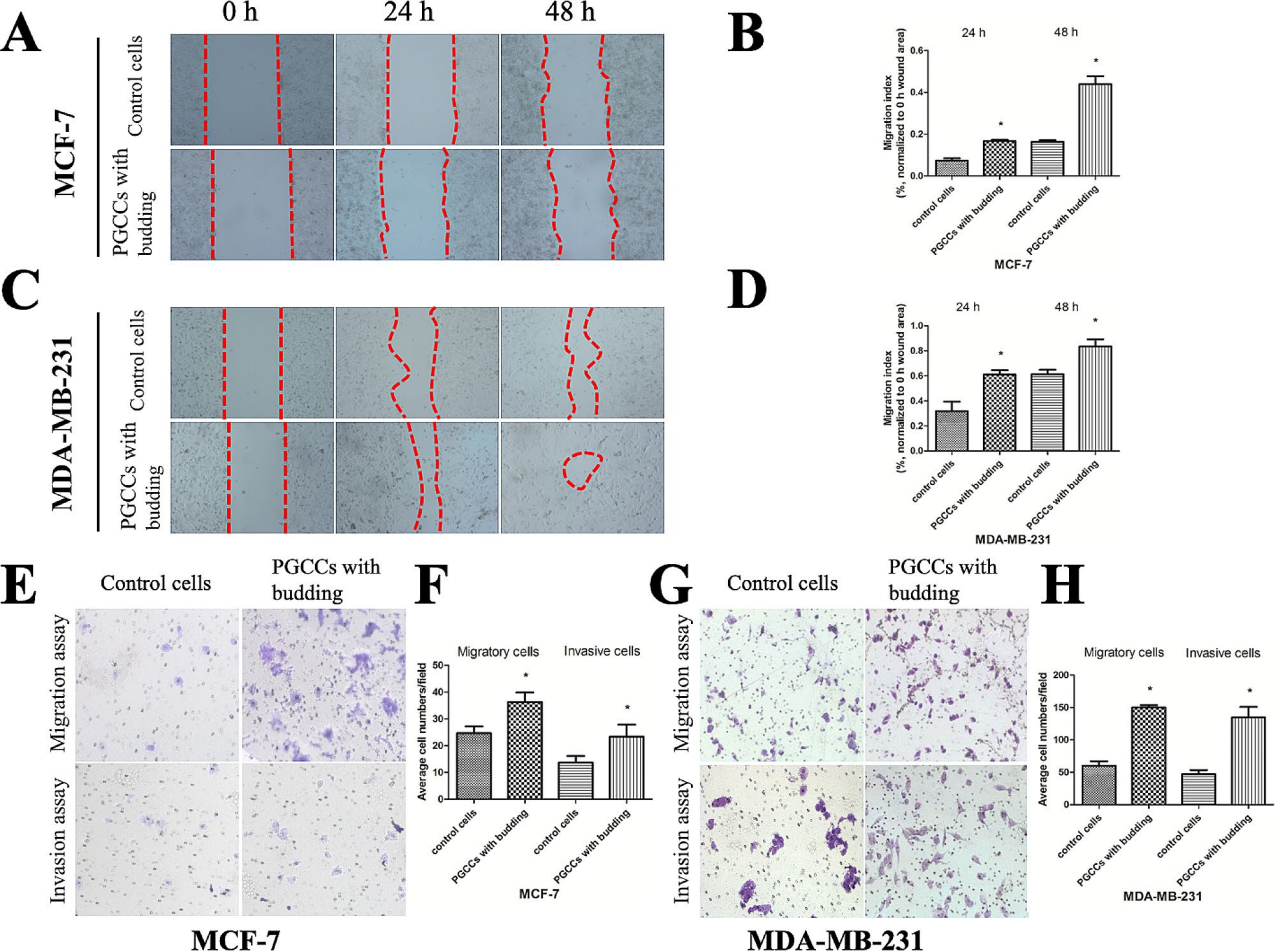
CoCl_2_ increases the migration and invasion of breast cancer cells. **a** Representative images of the wound-healing assay for MCF-7 cells at different times (×40). **b** MCF-7 cell migration is shown as a wound-healing index quantified by measuring at least three different wound areas. **c** Representative images of the wound-healing assay for MDA-MB-231 cells at different times (×40). **d** Quantitative data of MDA-MB-231 cell migration between control cells and PGCCs with budding. **e**,** g** Transwell migration and invasion assays were performed in control MCF-7 and MDA-MB-231 cells and PGCCs with budding (×100). Upper panels indicate the migration and lower panels show cell invasion. **f**,** h** Quantitative results of transwell migration and invasion assay in MCF-7 and MDA-MB-231 cells
