# Supplementary material for: The number of polyploid giant cancer cells and epithelial-mesenchymal transition-related proteins are associated with invasion and metastasis in human breast cancer
Source: J Exp Clin Cancer Res. 2015 Dec 24;34:158. doi: 10.1186/s13046-015-0277-8 (PMC4690326; doi:10.1186/s13046-015-0277-8)
Supplement: Additional file 1: Table-S1. — Detail information of patients in group I and group III. (DOC 27 kb) [file 13046_2015_277_MOESM1_ESM.doc]

Additional file 1: Table-S1. Detail information of patients in group I and group III.

|  | n | ER+  PR- | ER-  PR+ | ER+  PR+ | ER-  PR- | WHO grade | | | Lymph node metastasis number | | | Index of Ki-67 | | |
| --- | --- | --- | --- | --- | --- | --- | --- | --- | --- | --- | --- | --- | --- | --- |
|  |  |  |  |  |  | I | II | III | 1-3 | 4-9 | ≥10 | ≤10% | 11%-20% | >20% |
| Group I | 52 | 2 | 2 | 34 | 14 | 1 | 45 | 6 | 4 | 29 | 19 | 10 | 9 | 33 |
| Group III | 52 | 5 | 1 | 30 | 16 | 14 | 33 | 5 | 0 | 0 | 0 | 21 | 6 | 25 |
